# Inhibition of Ganglioside Synthesis Suppressed Liver Cancer Cell Proliferation through Targeting Kinetochore Metaphase Signaling

**DOI:** 10.3390/metabo11030167

**Published:** 2021-03-15

**Authors:** Ting Su, Xian-Yang Qin, Naoshi Dohmae, Feifei Wei, Yutaka Furutani, Soichi Kojima, Wenkui Yu

**Affiliations:** 1 Department of Intensive Care Unit, The Affiliated Drum Tower Hospital, Medical School of Nanjing University, Nanjing 210008, China; ting.su@riken.jp; 2 Liver Cancer Prevention Research Unit, RIKEN Cluster for Pioneering Research, Wako, Saitama 351-0198, Japan; yfurutani@riken.jp (Y.F.); skojima@postman.riken.go.jp (S.K.); 3 Biomolecular Characterization Unit, RIKEN Center for Sustainable Resource Science, Wako, Saitama 351-0198, Japan; dohmae@riken.jp; 4 Metabolomics Research Group, RIKEN Center for Sustainable Resource Science, Kanagawa, Yokohama 230-0045, Japan; feifei.wei@riken.jp

**Keywords:** ganglioside, liver cancer, spheroid, kinetochore, chromosome segregation, *N*-[2-hydroxy-1-(4-morpholinylmethyl)-2-phenylethyl]-decanamide, monohydrochloride, aurora kinase, proteome, lipidome, matrix-assisted laser desorption and ionization time-of-flight mass spectrometry

## Abstract

The incidence and mortality of liver cancer, mostly hepatocellular carcinoma (HCC), have increased during the last two decades, partly due to persistent inflammation in the lipid-rich microenvironment associated with lifestyle diseases, such as obesity. Gangliosides are sialic acid-containing glycosphingolipids known to be important in the organization of the membrane and membrane protein-mediated signal transduction. Ganglioside synthesis is increased in several types of cancers and has been proposed as a promising target for cancer therapy. Here, we provide evidence that ganglioside synthesis was increased in the livers of an animal model recapitulating the features of activation and expansion of liver progenitor-like cells and liver cancer (stem) cells. Chemical inhibition of ganglioside synthesis functionally suppressed proliferation and sphere growth of liver cancer cells, but had no impact on apoptotic and necrotic cell death. Proteome-based mechanistic analysis revealed that inhibition of ganglioside synthesis downregulated the expression of AURKA, AURKB, TTK, and NDC80 involved in the regulation of kinetochore metaphase signaling, which is essential for chromosome segregation and mitotic progression and probably under the control of activation of TP53-dependent cell cycle arrest. These data suggest that targeting ganglioside synthesis holds promise for the development of novel preventive/therapeutic strategies for HCC treatment.

## 1. Introduction

Liver cancer is a leading cause of cancer-related deaths (>600,000 death/year worldwide) with hepatocellular carcinoma (HCC) being the most common type [1]. Although the global cancer mortality has decreased during the last two decades, both the incidence and disease-specific mortality of liver cancer in both sexes have increased, constituting an urgent need to develop efficient and safe preventive and therapeutic treatments [2,3]. The increased incidence of HCC is particularly reported in Latin America and North America, where it is believed that non-alcoholic steatohepatitis (NASH), which is characterized by obesity-associated inflammation and a lipid-rich microenvironment, rather than viral hepatitis represents the leading etiology [4]. Lipogenesis is enhanced during liver tumorigenesis, which increases the risk of HCC by multiple mechanisms, such as providing structural components of cellular membranes and nutrient supply for cellular hyperproliferation [5,6]. There is also a growing focus on the role of lipid metabolism in regulating oncogenic signaling [7]. Membrane lipids function to define membrane domains, known as lipid rafts, and/or act as first and second messengers to recruit proteins from the cytosol and organize signal transduction [8]. Mature hepatocytes exhibit remarkable plasticity by direct dedifferentiation into an undifferentiated state in the chronic liver injury microenvironment, which are believed to represent the cells of origin, namely cancer stem cells (CSC), for liver cancer and are related to lipid metabolism reprogramming [9,10]. Therefore, understanding the changes and functions of lipid metabolism reprogramming during liver tumorigenesis will shed light on the development of novel therapeutic strategies for HCC treatment.

Gangliosides are a subtype of glycosphingolipids characterized by the presence of sialic acid residues. Gangliosides, such as monosialo-tetrahexosyl-ganglioside (GM) 1, are present in the external layer of plasma membranes and are known to be important in the organization of the membrane and membrane protein-mediated signal transduction [11,12]. As an excellent example, gangliosides are found in high concentrations in membrane lipid rafts [13], and play critical roles in regulating signal transduction involved in embryonic stem cell differentiation [14], neural development [15], and stemness maintenance [16,17]. In addition, the synthesis of gangliosides, such as disialo-ganglioside (GD) 3 and GD2, is increased in several types of cancers and is known to activate cancer cell signaling, resulting in increased cell proliferation and migration [18]. Therefore, targeting gangliosides holds promise for cancer therapy, including HCC [19,20].

In this study, we focused on the functional role of ganglioside synthesis in liver cancer cells using three analytic strategies. First, the expression of genes involved in ganglioside synthesis and the levels of GM1 ganglioside were determined in the livers of 3,5-diethoxycarbonyl-1,4-dihydrocollidine (DDC)-induced chronic liver injury mouse model, an animal model recapitulating the features of activation and expansion of liver progenitor-like cells [21,22]. The levels of GM1 ganglioside were compared between normal liver cells, liver cancer cells, and liver CSC-like cells. Second, functional analysis was performed using a chemical inhibitor of ganglioside synthesis, *N*-[2-hydroxy-1-(4-morpholinylmethyl)-2-phenylethyl]-decanamide, monohydrochloride (PDMP), to examine its role in the proliferation of HCC cells in monolayer and sphere cultures. Third, molecular mechanism analysis was performed using nano-scale liquid chromatographic tandem mass spectrometry (nLC-MS/MS)-based proteome analysis to explore the molecular targets of PDMP in HCC cells.

## 2. Results

### 2.1. Hepatic Ganglioside Synthesis Was Increased along with the Expansion of Mouse Hepatic Stem/Progenitor Cells

**Figure 1:**
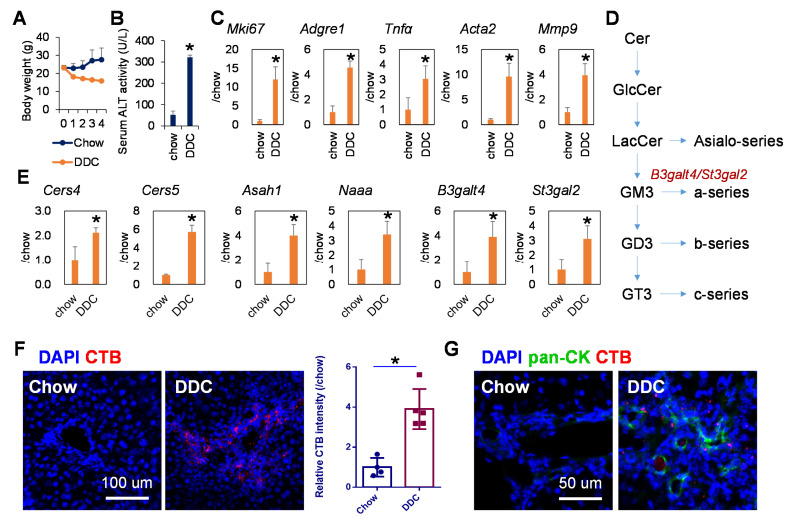
Enhanced ganglioside synthesis in the liver tissues of DDC-fed mice. (**A**) Changes in body weight of chow (n = 4) and DDC-fed mice (*n* = 5). (**B**) Serum ALT activity and (**C**) expression of cellular proliferation marker *Mki67*, inflammation-related genes *Adgre1* and *Tnfα*, and fibrosis-related genes *Acta2* and *Mmp9* in the liver tissues of mice fed with chow or DDC for 4 weeks. (**D**) Schematic overview of ganglioside synthesis pathway. Cer, ceramide; GlcCer, glucosylceramide; LacCer, lactosylceramide; GM3, monosialo-tetrahexosyl-ganglioside 3; GD3, disialo-ganglioside 3; GT3, trisialo-ganglioside 3. (**E**) Expression of ganglioside synthesis genes *Cers4*, *Cers5*, *Asah1*, *Naaa*, *B3galt4* and *St3gal2* in the liver tissues of mice fed with chow or DDC for 4 weeks. (**F**) Immunofluorescence staining of GM1 probe cholera toxin subunit B (CTB; red) in the liver of chow and DDC-fed mice (left), and the relative fluorescent intensity of CTB (right). Scale bar, 100 μm. (**G**) Immunofluorescence staining of liver progenitor cell marker pan-CK (green) and GM1 probe CTB (red) in the liver of chow and DDC-fed mice. Scale bar, 50 μm. Nuclei were stained with DAPI (blue). The data are presented as means ± SD. * *p* < 0.05 in Mann–Whitney U test.

Mice fed with 0.1% DDC-containing diet for 4 weeks demonstrated dramatic body weight loss (Figure 1A) and elevated serum alanine transaminase (ALT) activity (Figure 1B), the most common and best-established indicator of hepatocyte cell death [23]. In line with this, expression levels of genes related to compensatory cell proliferation (*Mki67)*, inflammation (*Adgre1* and *Tnfa)*, and fibrosis (*Acta2,* coding αSMA) and *Mmp9* were strongly increased in the livers of DDC-fed mice in comparison with those in chow-fed mice (Figure 1C). Next, the gene expression of enzymes involved in ganglioside synthesis was examined using PCR (Figure 1D). As shown in Figure 1E, the expression of ceramide synthases (*Cers4* and *Cers5*), acid ceramidase (*Asah1*), N-acylethanolamine acid amidase (*Naaa*), and beta-1,3-galactosyltransferase (*4B3galt4*) and β-galactoside α-2,3-sialyltransferase 2 (*St3gal2*), two key enzymes involved in the synthesis of GM1 ganglioside [24,25], was significantly upregulated in the livers of DDC-fed mice. In contrast, no obvious change or significant decrease in the gene expression of *Cers1* and *Cers2*, two enzymes that catalyze the synthesis of very long chain ceramides, were observed in the livers of DDC-fed mice, respectively (Figure S1). *Cers3* was not detected in mouse livers. To further explore the changes of ganglioside synthesis in the liver of DDC-fed mice, the levers of GM1 ganglioside in murine livers were measured by immunofluorescence staining with cholera toxin subunit B (CTB), which is a heat-labile enterotoxin and has been widely used as a molecular probe to detect binding to ganglioside GM1 due to its high binding affinity [26]. In accordance with the results of gene expression analysis, the level of CTB-defined ganglioside GM1 was dramatically increased in the livers of DDC-fed mice, especially in the portal vein region (Figure 1F). Furthermore, co-immunofluorescence staining demonstrated that the enhanced GM1 ganglioside was mainly observed in the region positive for pan-cytokeratin (pan-CK), which is a duct-specific marker used to define the ductal epithelium-like bi-potent characteristics of liver progenitor-like cells [27]. As a proof of concept, direct profiling of tissue lipids using matrix-assisted laser desorption and ionization time-of-flight mass spectrometry (MALDI-TOFMS) demonstrated that the contents of ceramide (d18:0/16:0, 504.513 *m/z*), GM1 ganglioside (18:1/18:0, 797.746 *m/z*), and glucosylceramides (d18:1/25:0, 848.765 *m/z*; d18:1/26:1, 870.741 *m/z*) were increased in the livers of DDC-fed mice than those in chow-fed mice (Figure S2). These data suggested that the increased ganglioside synthesis in DDC-induced liver injury was related to the progenitor response, which is critical for liver tumorigenesis [28]. Data mining of our previous human HCC transcriptome dataset [29,30] showed that the gene expression of *CERS2*, *CERS4*, *CERS5*, and *CERS6* was significantly increased in HCC tumors compared with that in non-tumor adjacent tissues, while no obvious difference was observed in *ASAH1* gene expression (Figure S3).

### 2.2. Ganglioside Synthesis Was Increased in Human Liver Cancer Stem-Like Cells

**Figure 2:**
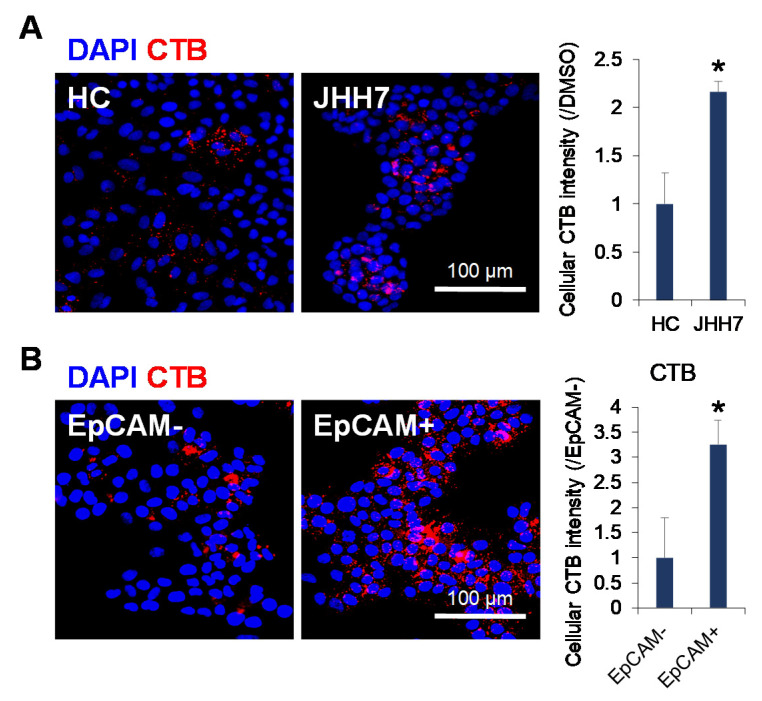
Enhanced ganglioside synthesis in human liver cancer stem-like cells. (**A**) Immunofluorescence staining of GM1 probe CTB (red) in normal hepatic cells HC and HCC cell line, JHH7 (left), and the relative fluorescent intensity of CTB (right). (**B**) Immunofluorescence staining of GM1 probe CTB (red) in sorted EpCAM-negative (EpCAM-) and EpCAM-positive (EpCAM+) HCC cell JHH7, and the relative fluorescent intensity of CTB (right). Scale bars, 100 μm. Nuclei were stained with DAPI (blue). The data are presented as means ± SD. * *p* < 0.05 in Student’s *t*-test.

To further explore the relationship between ganglioside synthesis and liver cancer, the level of GM1 ganglioside was evaluated using CTB staining in normal hepatic cell HC and HCC cell JHH7. Notably, JHH7 cells showed significantly higher GM1 ganglioside levels than those in HC cells (Figure 2A). Next, the correlation between GM1 ganglioside and pan-CK-positive liver progenitor-like cells in the livers of DDC-fed mice led us to examine whether ganglioside synthesis is altered in liver CSC-like cells, which can be identified based on their differentiation stage using specific markers such as EpCAM [31]. We previously isolated EpCAM^+^ cells from heterogeneous JHH7 cells using fluorescence-activated cell sorting and showed that these cells had features of epithelial-like CSCs with increased expression of lipid metabolism-related enzymes [30,32]. Here, we provide direct evidence that CTB staining-defined GM1 ganglioside levels were significantly higher in EpCAM^+^ CSC-like JHH7 cells than those in EpCAM^-^ non-CSC-like JHH7 cells (Figure 2B).

### 2.3. Inhibition of Ganglioside Synthesis Suppressed the Proliferation of Liver Cancer Cells

**Figure 3:**
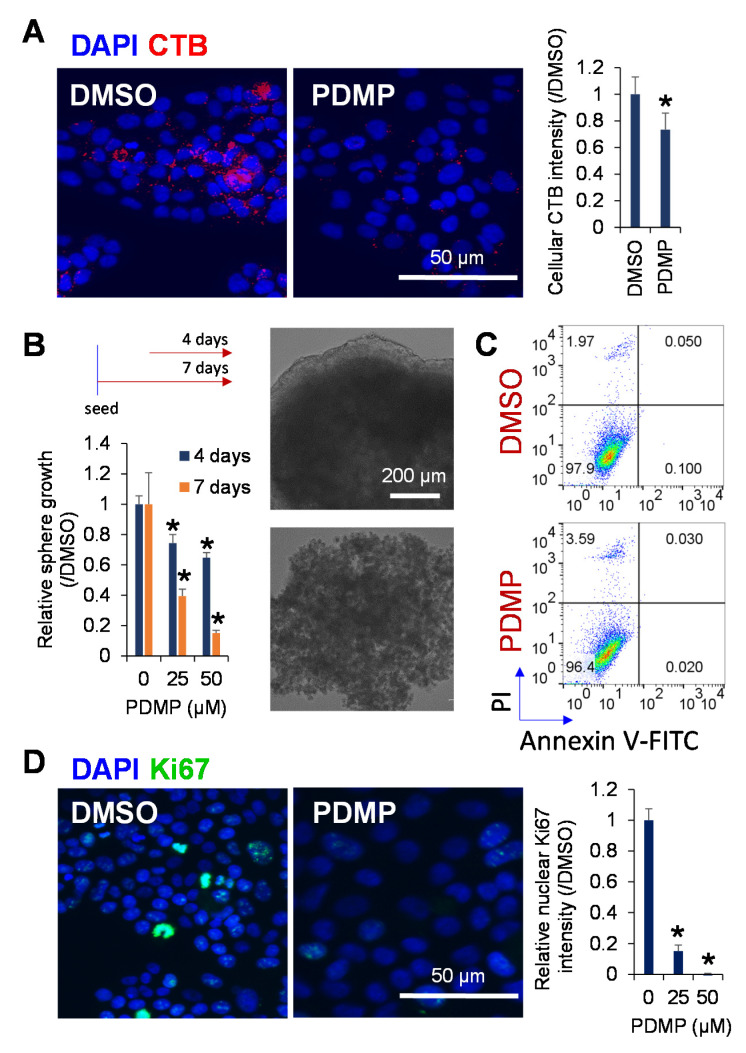
Inhibition of ganglioside synthesis suppressed spheroid proliferation of liver cancer cells. (**A**) Immunofluorescence staining of GM1 probe CTB (red) in the HCC cell line, JHH7, treated with vehicle control DMSO or ganglioside synthesis inhibitor PDMP at 50 μM for 2 h (left), and the relative fluorescent intensity of CTB (right). Scale bar, 50 μm. (**B**) Sphere proliferation of JHH7 cells treated with vehicle control DMSO or ganglioside synthesis inhibitor PDMP at indicated doses for 4 days or 7 days (left), and representative microscopic images of JHH7 cells grown in sphere cultures treated with vehicle control DMSO or ganglioside synthesis inhibitor PDMP at 50 μM for 7 days. Scale bar, 200 μm. (**C**) Apoptotic cell death of JHH7 cells grown in monolayer cultures treated with vehicle control DMSO or ganglioside synthesis inhibitor PDMP at 25 μM for 24 h was detected by dual staining with Annexin V-FITC and propidium iodide (PI) followed by flow cytometric analysis. (**D**) Immunofluorescence staining of cell proliferation marker Ki67 (green) in JHH7 cells grown in monolayer cultures treated with vehicle control DMSO or ganglioside synthesis inhibitor PDMP at 25 μM for 24 h (left), and the relative fluorescent intensity of nuclear Ki67 (right). Nuclei were stained with DAPI (blue). Scale bar, 50 μm. The data are presented as means ± SD. * *p* < 0.05 in Student’s *t*-test.

Next, we examined whether the inhibition of ganglioside synthesis using a chemical inhibitor PDMP might play a functional role in the proliferation of JHH7 cells in both monolayer and sphere cultures. According to CTB staining, PDMP treatment efficiently reduced GM1 ganglioside synthesis in JHH7 cells (Figure 3A). In a 3D culture system as a mimic of the tumor microenvironment enriched with CSC-like cells [33], PDMP treatment inhibited the spheroid proliferation of JHH7 cells in a dose- and time-dependent manner (Figure 3B). These data are in agreement with the above observations that ganglioside synthesis was increased in CSC-like JHH7 cells and support ganglioside as an important player in maintaining the sphere growth of HCC cells. Unexpectedly, flow cytometry analysis demonstrated that the proportion of Annexin V-positive apoptotic cells was not affected, while the proportion of propidium iodide (PI)-positive necrotic cells (1.97% and 3.59% in dimethyl sulfoxide (DMSO) and PDMP-treated cells, respectively) was only slightly increased by PDMP treatment in JHH7 cells in monolayer cultures (Figure 3C). In contrast, immunofluorescence staining showed that the percentage of cells expressing the cell proliferation marker Ki67 was almost completely suppressed in PDMP-treated JHH7 cells in monolayer cultures (Figure 3D). Collectively, these data indicate that ganglioside is critical in the control of cell proliferation and sphere growth of HCC cells, and inhibition of ganglioside synthesis suppressed cell cycle progression but did not induce cell death in HCC cells.

### 2.4. Inhibition of Ganglioside Synthesis Affected the Lipid Composition of Liver Cancer Cells

Given the critical role of ganglioside as a component of lipid membrane domains, we investigated whether the inhibition of ganglioside synthesis had an impact on the membrane lipid composition of JHH7 cells. MALDI-TOFMS is a powerful technology for detecting the fatty acyl composition of lipids in a very short operation time [34]. Lipids were extracted from JHH7 cells treated with DMSO or PDMP at increasing concentrations for 24 h and then subjected to MALDI-TOFMS analysis. Principle component analysis (PCA), a standard technique of pattern recognition and multivariate data analysis, was applied to the MS spectra. The scores plot (PC 1 vs. 2) discriminated lipid composition of DMSO and PDMP-treated JHH7 cells in a dose-dependent manner, while PC1 explained as high as 65.8% of the total variance (Figure S4). Given that differences were identified using an unsupervised analysis, without any prior information about the samples, the observed discrimination demonstrates that the inhibition of ganglioside synthesis with PDMP is strongly represented by the change in lipid profiles in HCC cells.

### 2.5. Proteome-Based Analysis of Downstream Signaling Pathway Following Ganglioside Synthesis Inhibition in Liver Cancer Cells

**Figure 4:**
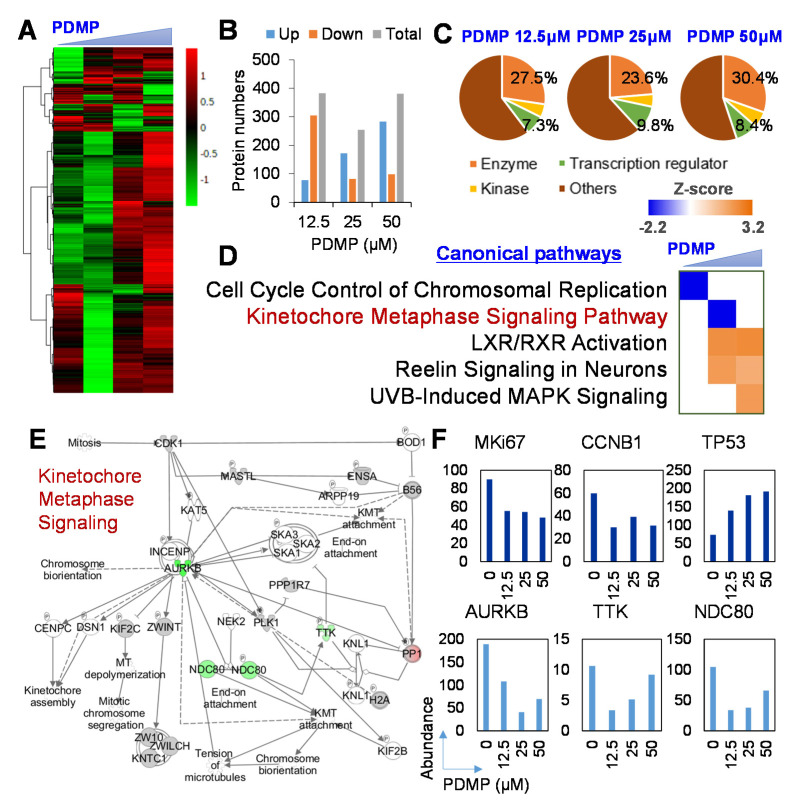
Proteome-based analysis of downstream signaling pathway following ganglioside synthesis inhibition in liver cancer cells. nLC-MS/MS-based proteome analysis was performed in the HCC cell line, JHH7, treated with vehicle control DMSO or ganglioside synthesis inhibitor PDMP at 12.5, 25, and 50 μM for 24 h. (**A**) Heatmap visualization of a total of 4828 identified proteins. (**B**) Summary of the number and (**C**) functional annotation of differentially expressed proteins in JHH7 cells treated with PDMP in comparison with those treated with DMSP with a cut-off of fold change more than 2. (**D**) Canonical pathway analysis of differentially expressed proteins generated using the knowledge-based functional analysis software Ingenuity Pathways Analysis (IPA) with a cut-off of z-score more than 2. (**E**) A representative network related to cell cycle progression entitled “Kinetochore Metaphase Signaling”. Upregulated proteins under the control of PDMP are shown in red, downregulated proteins under the control of PDMP are shown in green, and proteins that were not annotated in this study but were part of this network, are shown in white. (**F**) Expression of enriched proteins involved in the kinetochore metaphase signaling pathway and cell cycle related proteins MKi67, CCNB1, and TP53 detected by nLC-MS/MS.

Next, to explore the molecular mechanism underlying the effects of ganglioside synthesis inhibition by PDMP on the proliferation of JHH7 cells, proteome analysis was performed to examine the global protein expression changes. A total of 4828 proteins were successfully detected in JHH7 cells using nLC-MS/MS analysis (Table S1). Hierarchical clustering demonstrated diverse protein expression profiles of JHH7 cells treated with PDMP at increasing concentrations (Figure 4A). PCA analysis of the protein expression profiles showed a similar distribution to that of the MALDI-TOFMS-based lipidome analysis (Figure S5). Proteins with a cut-off fold change of more than 2 in comparison to that of DMSO were selected for further analysis. The number of differentially expressed proteins at each concentration of PDMP treatment was comparable (Figure 4B). Furthermore, PDMP treatment at different concentrations showed similar functional annotations of differentially expressed proteins (Figure 4C). The highest percentage of differentially expressed transcriptional factors, approximately 9.8%, was observed with PDMP treatment at 25 μM. To further understand the biological process under the control of PDMP, the differentially expressed proteins were imported into the IPA program. An important statistical measure of IPA pathway analysis is the activation z-score, which can be used to identify likely regulating molecules based on a statistically significant pattern match of up- and down-regulation, and also to predict the activation state (either activated or inhibited) of a putative regulator [35]. Canonical pathway analysis of IPA showed that cell cycle progression-related signaling pathways, the “Cell Cycle Control of Chromosomal Replication” and the “Kinetochore Metaphase Signaling Pathway”, were functionally inhibited by PDMP treatment at 12.5 μM and 25 μM, respectively (Figure 4D). These concentrations also showed the most diverse lipid and protein profiles in comparison with that of the DMSO control (Figures S4 and S5). Detailed analysis of the schematic pathway of “Kinetochore Metaphase Signaling” indicated that three critical regulators of alignment and segregation of chromosomes during mitosis, aurora B kinase (AURKB), dual specificity protein kinase TTK (TTK, also referred to as Mps1), and kinetochore protein NDC80 homolog (NDC80) were under the control of PDMP in JHH7 cells (Figure 4E). Relative protein quantification by nLC-MS/MS analysis showed that the relative abundances of AURKB, TTK, and NDC80 were decreased by PDMP in JHH7 cells (Figure 4F). In line with this, the relative abundance of Ki67, a cell proliferation marker, and that of CCNB1, a regulatory protein involved in mitosis, were also decreased by PDMP in JHH7 cells (Figure 4F). In contrast, the relative abundance of TP53, a tumor suppressor protein that regulates the expression of target genes involved in cell cycle arrest and apoptosis in response to diverse cellular stresses, was increased by PDMP in JHH7 cells (Figure 4F).

### 2.6. Inhibition of Ganglioside Synthesis Targets Chromosome Segregation Signaling Pathway in Liver Cancer Cells

**Figure 5:**
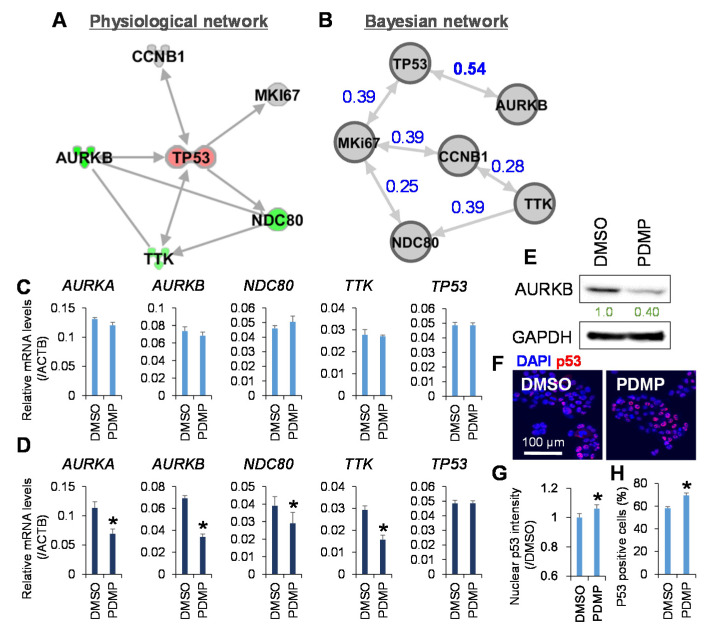
Inhibition of ganglioside synthesis targets kinetochore metaphase signaling in liver cancer cells. (**A**) Physiological network generated using the Ingenuity Knowledge Base of the IPA software. (**B**) Bayesian network generated using the score-based hill-climbing learning algorithm in the bnlearn R package and visualized using Gephi software. Expression of genes involved in kinetochore metaphase signaling pathway in the HCC cell line, JHH7, treated with vehicle control DMSO or ganglioside synthesis inhibitor PDMP at 25 μM for 4 h (**C**) and 24 h (**D**) using real-time PCR. (**E**) Western blot analysis of AURKB and GAPDH in JHH7 cells treated with vehicle control DMSO or 25 μM PDMP for 24 h. Band intensities were quantified using Image J software. The protein level of AURKB was normalized to GAPDH and expressed as fold change compared with vehicle control DMSO set as 1. (**F**) Immunofluorescence staining for p53 in JHH7 cells treated with vehicle control DMSO or 25 μM PDMP for 24 h. Scale bar, 100 μm. (**G**) The relative fluorescent intensity of nuclear p53 protein vs. vehicle control DMSO. (**H**) Percentages of p53 positive cells among the total number of JHH7 cells counted. The data are presented as means ± SD. * *p* < 0.05 in Student’s *t*-test.

Finally, network analysis was applied to determine further the connection between the molecular targets of PDMP involved in kinetochore metaphase signaling and cell cycle regulators. The physiological network generated based on findings of previous studies using the Ingenuity Knowledge Base showed that TP53 played a central role in the inferred network (Figure 5A). In accordance with this, the Bayesian network showed that the node of TP53 was located at the top of the network hierarchy, and the strongest connection was observed between TP53 and AURKB (Figure 5B). The Bayesian network represents the joint posterior probability distribution over the whole set of variables in a system and is considered more biologically interpretable because of the removal of indirect correlations [36]. Intriguingly, upstream regulator analysis, the causal analytics algorithms in IPA, revealed that TP53 was the only upstream transcription regulator of AURKB, whose expression was regulated by PDMP in JHH7 cells (Figure S6). Finally, PCR analysis was performed to determine whether PDMP regulates kinetochore metaphase signaling at the transcriptional level. No obvious effect was observed in JHH7 cells treated with 25 μM PDMP for 4 h (Figure 5C). However, the gene expression levels of *ARUKA*, *TTK*, and *NDC80* were significantly reduced in JHH7 cells treated with 25 μM PDMP for 24 h (Figure 5D). In line with this, the protein levels of AURKB were dramatically reduced in JHH7 cells treated with 25 μM PDMP for 24 h (Figure 5E). In contrast, no effect of PDMP treatment was observed on TP53 gene expression in JHH7 cells treated for either 4 h or 24 h (Figure 5C,D). Finally, we examined the nuclear accumulation of p53 protein, which is an important event of p53 activation in stressed cells [37]. Western blotting analysis of cellular fractions showed that the protein levels of nuclear p53 were slightly increased in JHH7 cells treated with 25 μM PDMP for 24 h (Figure S7). Similarly, immunofluorescence staining showed that both the protein levels of nuclear p53 and the percentage of p53-positive cells were slightly but significantly increased in JHH7 cells treated with 25 μM PDMP for 24 h (Figure 5F–H).

## 3. Discussion

Our study showed that inhibition of ganglioside synthesis suppressed cell proliferation and sphere growth of HCC cells, partly by targeting key regulators of mitosis, such as AURKB, TTK, and NDC80, which are involved in kinetochore metaphase signaling. The kinetochore is a large protein complex linking the centromeric DNA to microtubules of mitotic spindles and is responsible for mediating chromosome segregation during meiosis and mitosis [38]. Dysregulation of kinetochores, such as merotelic kinetochore orientation, is defined as a critical source of chromosome instability, which is a hallmark of cancer that contributes to tumor heterogeneity and malignancy [39,40]. In line with this, increased expression of kinetochore genes has been correlated with adverse tumor characteristics and poor prognosis, making it an attractive target for cancer therapeutics [41]. Indeed, the chemical inhibitors of the kinetochore genes AURKB [42], TTK [43], and NDC80 [44] have been proven effective in cancer therapy in preclinical studies. In addition, inhibition of ganglioside synthesis by PDMP also suppressed the gene expression of aurora kinases *AURKA* and *AURKB*. Beyond the kinase-dependent regulatory roles in entry into mitosis, there is growing attention on aurora kinase inhibitors in mediating the proteolytic degradation of undruggable oncogenes such as MYC family proteins [45,46]. Of interest, a combined effect was observed by targeting GD2 ganglioside and AURKA in the inhibition of MYCN expression and induction of cell death in neuroblastoma cells [47] Collectively, these data suggest that targeting ganglioside synthesis is a promising therapeutic strategy for liver cancer.

Time course analysis showed that PDMP treatment for 24 h but not 4 h suppressed the gene expression of *AURKA*, *AURKB*, *TTK,* and *NDC80* in JHH7 cells, indicating an indirect mechanism through ganglioside-mediated signaling pathways. Further upstream regulatory analysis in IPA and Bayesian network analysis on proteome profiling identified TP53 as the upstream transcription regulator of kinetochore genes regulated by PDMP in JHH7 cells. Indeed, TP53 is directly involved in the repression of *AURKB* gene expression in the cell cycle arrest of human prostate cancer cells [48]. Although the protein abundance of TP53 was increased by PDMP in JHH7 cells, no effect was observed on the gene expression of *TP53*, suggesting post-transcriptional regulation of TP53 by PDMP. Somatic mutations in TP53 are regarded as a hallmark of cancer because mutant p53 proteins not only lose tumor suppressive activities but often gain oncogenic function [49]. The expression of alpha-N-acetylneuraminide alpha-2,8-sialyltransferase (*ST8SIA1*), a key enzyme regulating GD2 ganglioside synthesis, was positively correlated with mutations in p53 in human breast cancers [50]. Knockout of *ST8SIA1* expression almost completely inhibited the tumor formation activity of breast cancer cells, accompanied by the activation of tumor suppressor phosphatase and tensin homolog deleted on chromosome 10 (PTEN) and TP53 signaling pathways [50]. In addition, a monoclonal anti-GD2 antibody, 14G2a, against neuroblastoma induced the activation of p53 in neuroblastoma cells [47,51]. These data suggest that inhibition of ganglioside synthesis suppresses the proliferation of HCC cells by inhibiting the alignment and segregation of chromosomes, which is probably under the control of activation of TP53-dependent cell cycle arrest.

Unexpectedly, inhibition of ganglioside synthesis by PDMP dramatically suppressed the cell proliferation and sphere growth of JHH7 cells, but none of the two major forms of cell death, apoptosis and necrosis, was observed. This could be partly explained by further mechanistic analysis revealing that PDMP exerted its growth-suppressing activity through downregulation of kinetochore gene expression and the consequent chromosome segregation and mitotic progression. Dying cells release damage-associated molecular patterns (DAMPs) such as high-mobility group box 1 (HMGB1) and ATP, most of which are recognized by pattern recognition receptors in immune cells [23]. DAMP-induced sterile inflammation may trigger liver fibrosis [52] and has been related to CSC activation and tumorigenesis in chronic liver disease [53]. Therefore, DAMPs released from dying cells in cancer therapy are believed to play detrimental roles in the promotion of tumor metastasis, progression, and resistance to anticancer treatments [54]. Therefore, it is rational to propose that targeting ganglioside synthesis by PDMP might be a beneficial option for liver cancer therapy.

Although this study provided a general view of the role of ganglioside synthesis in the control of HCC cell proliferation, there are some limitations that might serve as future research avenues. First, further study is warranted to explore the upstream regulatory mechanism of ceramide and ganglioside synthesis during hepatic tumorigenesis. The composition of membrane lipid species is complicated and dynamically organized in a circular network [55]. Both the genes encodings enzymes involved in ceramide synthesis (*Cers4* and *Cers5*) and degradation (*Asah1*) were upregulated in the livers of DDC-fed mice. In contrast, the gene expression of *CERS4* and *CERS5*, but not *ASAH1*, was significantly increased in HCC tumors than that in non-tumor adjacent tissues. Although ASAH1 is well-known in regulating the breakdown of ceramide [56,57], an unexpected reduction in ceramide was observed following knockdown of mouse *Asah1* mRNA by shRNA in murine macrophage RAW 264.7 cells [55]. In line with this, a reverse activity of ASAH1 in promoting ceramide synthesis was also reported [58]. The diverse expression pattern of ASAH1 in human HCC tumors and the livers of DDC-fed mice with cholestatic liver injury indicated that ceramide metabolism might be regulated in cell type and tissue context-dependent manners. In addition, hepatic ceramide levels and acid ceramidase activity transiently increased during murine hepatic ischemia reperfusion injury, indicating a dynamic balance between the generation and metabolism of ceramide [59]. Second, although Western blotting and immunofluorescence staining showed that the protein levels of nuclear p53 were increased by PDMP in JHH7 cells, the change in p53 protein expression was slight. Further study is needed to explore the mechanism of p53 activation or other p53-independent mechanisms under the control of PDMP. Finally, although this study focused on the role of ganglioside synthesis, other sphingolipids should also be considered in future studies. Gene expression analysis in both the livers of DDC-fed mice and human HCC tumors showed the increase in gene expression of ceramide synthases such as *CERS4* and *CERS5*. In addition, PDMP is a classic glucosylceramide synthase and lactosylceramide synthase inhibitor targeting beta-1,4-galactosyltransferase 5/6 (B4GALT5/6), which is critical in regulating the biosynthesis of lactosylceramide, a common precursor of lactose series of glycosphingolipids, including gangliosides [60]. PDMP prevented the conversion of ceramide into glycosylated ceramides such as glucosylceramide [61], lactosylceramide [61], monosialo gangliosides GM1 [62] and GM3 [63,64], and disialo ganglioside [61], while induced the content of ceramide [61,65,66] or had limited effects on its conversion into sphingomyelin [67,68] and sphingosine [69]. Ceramide as the central molecule of sphingolipid metabolism has been reported to play a pro-apoptotic and anti-proliferative role in chronic liver diseases such as NASH [70], alcoholic steatohepatitis [71], and hepatic ischemia reperfusion injury [59]. Recently, the turnover from ceramide to its downstream sphingolipids such as sphigosine-1-phosphate emerge as novel diagnostic biomarkers and therapeutic targets for HCC [72].

## 4. Materials and Methods

### 4.1. Materials

The GM1 probe Alexa Fluor 647 conjugated CTB was obtained from Thermo Fisher Scientific (C34778; Waltham, MA, USA). The ganglioside synthesis inhibitor PDMP was purchased from Cayman Chemical (62595; Ann Arbor, MI, USA). DDC (137030), 2,5-Dihydroxybenzoic acid (DHB; 149357), trans-2-[3-(4-tert-Butylphenyl)-2-methyl-2-propenylidene]malononitrile (DCTB; 727881), cesium triiodide (483338), acetonitrile (271004), and trifluoroacetic acid (A2889) were purchased from Sigma-Aldrich (St. Louis, MO, USA). A standard rodent chow diet CE-2 containing 0.1% wt/wt DDC was prepared by Japan Clea (Tokyo, Japan).

### 4.2. Animal Experiments

Animal experiments were performed in accordance with protocols approved by the Institutional Committee of Animal Experiment of RIKEN (W2020-2-001) and adhered to the guidelines of the Institutional Regulations for Animal Experiments and Fundamental Guidelines for Proper Conduct of Animal Experiments and Related Activities in Academic Research Institutions under the jurisdiction of the Ministry of Education, Culture, Sports, Science and Technology, Japan. Male C57BL6/J mice (age 6 weeks) were housed under constant temperature (22 ± 1 °C) with free access to food and water. To induce chronic liver injury and liver progenitor-like cell activation and expansion, mice were fed a DDC-diet for 4 weeks.

### 4.3. Serum ALT Activity

Serum samples were diluted 1:2 with PBS, and ALT activity levels were examined using the GPT-JS kit (Denka Seiken, Tokyo, Japan) in accordance with the manufacturer’s instructions. The absorbance was measured at 340 nm using a plate reader (EnSight, PerkinElmer Inc., Waltham, MA, USA).

### 4.4. RNA Isolation and Real-Time PCR

Total RNA was isolated from mouse liver tissues or human cell cultures using a FastGene RNA Basic Kit (FG-80250, NIPPON Genetics, Tokyo, Japan). RNA quantification was performed using a NanoDrop spectrophotometer (NanoDrop Products, Wilmington, DE, USA). cDNA was synthesized using a PrimeScript RT Master Mix Kit (TaKaRa Bio, Otsu, Japan). PCR reactions were performed using a Roche LightCycler 96 Real-Time PCR System (Roche Diagnostic Co., Ltd., Tokyo, Japan) and SYBR Premix ExTaq II (TaKaRa Bio). Primer sequences are listed in Table 1. Gene expression was normalized to that of *16s* using the ΔΔCT method and set to 1 for the control chow-fed mice.

### 4.5. Immunofluorescence Staining

For the liver tissues, frozen sections (10 μm) were cut and mounted on slides and dried using a dryer prior to use. Cryosections were washed with PBS containing 0.1% Tween 20 (PBST), fixed with 4% paraformaldehyde (PFA) for 10 min at room temperature, and washed twice with PBST for 5 min. Liver sections were heated in Target Retrieval Solution (DAKO Corporation, Carpinteria, CA, USA) in a microwave for 1 min for antigen retrieval. After washing twice with PBST for 5 min, the sections were permeabilized with PBS containing 0.3% Triton X-100 for 10 min at room temperature and incubated in blocking buffer (5% normal goat serum in PBS containing 0.3% Triton X-100) for 30 min in a humidified chamber at room temperature. Alexa Fluor 647 conjugated CTB (1:1000 dilution) or rat anti-cytokeratin pan type I/II antibody cocktail (pan-CK, 1:100 dilution, 151202, BioLegend, San Diego, CA, USA) were diluted in blocking buffer and incubated with the sections overnight at 4 °C. On the next day, the sections were stained with donkey anti-rat Alexa Fluor 488-conjugated secondary antibody (1:500 dilution; Invitrogen, Grand Island, NY, USA) for 1 h at room temperature. The sections were mounted with 4′,6-diamidino-2-phenlindole (DAPI; 1:2000 dilution; Wako Industries, Osaka, Japan) in blocking buffer for 30 min at room temperature. Immunofluorescence staining signals were detected and quantified with a Zeiss LSM 700 laser scanning confocal microscope (Carl Zeiss Inc., Jena, Germany). For cell cultures, the cells were fixed with 4% PFA for 10 min and incubated with 0.1% Triton X-100 in PBS for 10 min at room temperature. After blocking with 5% FBS-PBS for 1 h at room temperature, cells were incubated with rat monoclonal anti-Ki67 antibody (1:200 dilution, 151202, BioLegend, San Diego, CA, USA) or rabbit anti-p53 antibody (1:200 dilution, 9282, Cell Signaling Technology, Danvers, MA, USA) overnight at 4 °C. The cells were then washed and stained with donkey anti-rat Alexa Fluor 488 or donkey anti-rabbit Alexa Fluor 555-conjugated secondary antibodies (1:500, Invitrogen), and nuclei were visualized by DAPI staining (Wako Industries). Immunofluorescence staining signals were detected with an ImageXpressMICRO High Content screening System (Molecular Devices, Sunnyvale, CA, USA), and morphological analysis was performed using MetaXpress Image Analysis software (Molecular Devices).

### 4.6. Cell Culture

The normal hepatic cell line, HC, was purchased from Cell Systems (Kirkland, WA, USA). The HCC cell line JHH7 was kindly provided by Prof. T. Matsuura of the Jikei University School of Medicine, Tokyo, Japan [73]. The cells were maintained at 37 °C and 5% CO_2_ in Dulbecco’s Modified Eagle Medium (DMEM, Wako Industries, Osaka, Japan) containing 10% fetal bovine serum (FBS; Mediatech, Herndon, VA, USA), 100 U/mL penicillin/streptomycin, and 2 mM _L_-glutamine (Mediatech, Herndon, VA, USA).

### 4.7. Cell Sorting

The expression of CSC marker EpCAM was analyzed, and EpCAM positive and negative subpopulations were isolated using FACS as previously described [30].

### 4.8. 3D Spheroid Cultures

3D spheroid cultures were performed on non-adherent 96-well round-bottomed Sumilon PrimeSurface™ plates (MS-9096U, Sumitomo Bakelite, Tokyo, Japan) as previously described [32]. The spheroids were grown for 4 days, then 50 µL of media was exchanged with 50 µL fresh serum-free media containing 10 µM CAY10566 and/or 100 µM fatty acids. The spheroids were further cultured for 7 days, and photographs were taken using an optical microscope (DS-Fi1, NIKON, Tokyo, Japan).

### 4.9. Spheroid Proliferation Assay

Spheroid proliferation was measured using the CellTiter-Fluor™ Cell Viability Assay (Promega Corporation, Madison, WI, USA) as previously described [32]. Fluorescence was measured using a plate reader (ARVO MX, Perkin Elmer Inc., Waltham, MA, USA).

### 4.10. Annexin V Staining Assay

Apoptotic cells were detected using the FITC Annexin V Apoptosis Detection Kit with propidium iodide (PI) (640914, BioLegend, San Diego, CA, USA). Briefly, the cells were washed with cell staining buffer (420201, BioLegend, San Diego, CA, USA) and resuspended in Annexin binding buffer. The cells were then labeled with Annexin V-FITC and PI at room temperature for 15 min in the dark. The fluorescence of stained cells was measured using a BD LSR flow cytometer and CellQuest Pro software (BD Biosciences, San Jose, CA, USA). The data were further analyzed using FlowJo software (Tree Star, Inc., Ashland, OR, USA).

### 4.11. Lipid Extraction

Lipids were isolated from HCC cells JHH7 treated with vehicle control DMSO or ganglioside synthesis inhibitor PDMP at 12.5, 25, and 50 μM for 24 h using the chloroform/methanol/water extraction method as previously described [74]. The chloroform phase containing lipids was evaporated under vacuum in SpeedVac (CC-105, TOMY, Tokyo, Japan) and reconstituted in 50 μL isopropanol (Wako Industries, Tokyo, Japan). The extracted lipids were stored at −80 °C with inert nitrogen for further use.

### 4.12. MALDI-TOFMS-Based Lipidomic Analysis

Lipids were mixed with 15 mg/mL DHB matrix (1:5 *v*/*v*) in 90% acetonitrile/0.1% trifluoroacetic acid aqueous solution, and 0.5 μL of the mixture was loaded onto a MALDI-TOF target plate (MTP 384 target plate ground steel, Bruker Daltonics, Leipzig, Germany). For direct profiling of tissue lipids, frozen liver sections (10 μm) were cut and mounted on ITO-coated glass slides (Bruker Daltonics) and dried in a vacuum desiccator prior to use. Matrix deposition was performed using the nozzle spraying method with the TM Sprayer (HTX Technologies, Carrboro, NC, USA). Mass spectrometric analysis was performed using a rapifleX MALDI Tissuetyper mass spectrometer (Bruker Daltonics) at a spatial resolution of 20 µm. Peak calibration was carried out with a mixture of 10 mg/mL DCTB and 1 mg/mL cesium triiodide (1:1 *v*/*v*). Each collected spectrum was the sum of 10 single spectra in the range between 500 and 2000 Da obtained by shooting the laser at random positions on the target spot. Data were analyzed using FlexImaging software (Bruker Daltonics). Peak intensity was normalized to the protein concentration. Fore mass spectrometry imaging, peak assignment was performed by LC-MS searches in the Human Metabolome Database (http://www.hmdb.ca/; 25 February 2021) using positive ion mode and the threshold of 10 ppm. The identified peaks of sphingolipids were visualized using SCiLS Lab software (Bruker Daltonics).

### 4.13. nLC-MS/MS-Based Proteomic Analysis

JHH7 cells treated with vehicle control DMSO or ganglioside synthesis inhibitor PDMP at 12.5, 25, and 50 μM for 24 h were lysed using RIPA buffer and protein concentration was measured with the Pierce BCA Protein Assay Kit (Thermo Fisher Scientific). After boiling at 98 °C for 10 min, the protein samples were purified and digested using the filter-aided sample preparation method [75]. nLC-MS/MS analysis was performed using EASY-nLC 1000 (Thermo Fisher Scientific, Inc., San Jose, CA, USA) and Q Exactive mass spectrometer (Thermo Fisher Scientific) equipped with a NANO HPLC capillary column C18 (0.075 mm ID × 150 mm length, 3 µm particle size, Nikkyo Technos, Tokyo, Japan) using a linear gradient (120 min, acetonitrile/0.1% formic acid) at a flow rate of 300 nL/min. The resulting MS and MS/MS data were searched against the Swiss-Prot database using Proteome Discoverer (Ver.2.2, Thermo Fisher Scientific) with MASCOT search engine software (Ver. 2.7.0, Matrix Science, London, UK).

### 4.14. Knowledge-Based Pathway Analysis

To explore the biological interpretation of the proteome data, the canonical pathway was identified using the knowledge-based functional analysis software Ingenuity Pathways Analysis (IPA, Ingenuity Systems, Mountain View, CA, USA) as previously described [76]. An absolute z-score of more than 2 was considered significant.

### 4.15. Western Bloting

The cells were lysed using RIPA buffer buffer. For subcellular protein fractionation, the cells were lysed in the fractionation buffer (250 mM sucrose, 50 mM Tris-HCl, pH 7.4, 5 mM MgCl_2_, 1 mM EDTA, 1 mM EGTA) and centriguated at 300 g for 5 min. The supernant was collected as cytoplasmic protein fraction. The nuclear pellet was resuspended in fractionation buffer containing 1% Triton X-100. Protein concentration was measured with the Pierce BCA Protein Assay Kit (Thermo Fisher Scientific). After a brief sonication and boiling at 95 °C for 5 min, the protein samples were resolved by sample buffer for SDS/PAGE, run on a 5–20% gradient gel, e-PAGE (EHR-R520L, Atto, Tokyo, Japan) and transferred to a PVDF membrane (Bio-Rad Laboratories, Hercules, CA, USA). The membranes were blocked with 5% nonfat dry milk in Tris-buffered saline and 0.1% Tween and then were probed with rabbit anti-p53 (1:1000 dilution; 9282, Cell Signaling Technology), rabbit anti-AURKB (1:1000 dilution; 3094, Cell Signaling Technology), rat anti-human GAPDH (1:1000 dilution; 607902, BioLegend), and rabbit anti-human Lamin B1 (1:1000 dilution; ab16048, Abcam, Cambridge, MA, USA) antibodies overnight at 4 °C. The blots were then incubated with HRP-conjugated anti-rabbit or anti-rat secondary antibodies (1:2000 dilution) and detected using the Amersham ECL Plus Western Blotting Detection System (GE Healthcare, Piscataway, NJ, USA). Band intensities were quantified using Image J software (National Institutes of Health, Bethesda, MD, USA).

### 4.16. Multivariate and Statistical Analysis

Unsupervised principal component analysis (PCA) and Bayesian network analysis were performed on the R platform. Bayesian network inference was performed with the score-based hill-climbing learning algorithm. The network was visualized using the Gephi software (https://gephi.org/; 25 February 2021). Quantitative data are expressed as the mean ± SD of at least three biological replicates. The significance of differences between values was assessed using the Mann-Whitney U test or Student’s *t*-test in Excel or Graph Prism. A *p*-value < 0.05 was considered significant.

## 5. Conclusions

In summary, this study provided evidence that ganglioside synthesis was increased in the livers of DDC-fed mice with expansion of liver progenitor-like cells and in liver cancer (stem) cells. Chemical inhibition of ganglioside synthesis by PDMP functionally suppressed cell proliferation and sphere growth of liver cancer cells, but had no impact on apoptotic and necrotic cell death. Proteome-based mechanistic analysis revealed that PDMP exerted its growth-suppressing activity through downregulation of kinetochore gene expression and the consequent chromosome segregation and mitotic progression, which is probably under the control of activation of TP53-dependent cell cycle arrest. These data suggest that targeting ganglioside synthesis holds promise for the development of novel preventive/therapeutic strategies for HCC treatment.

## Figures and Tables

**Table 1:** Primer sequences used in this study.

Gene Symbol	Forward	Reverse
Mouse		
*16s*	AGGAGCGATTTGCTGGTGTGGA	GCTACCAGGGCCTTTGAGATGGA
*Mki67*	AGGGTAACTCGTGGAACCAA	TTAACTTCTTGGTGCATACAATGTC
*Adgre1*	TGTCTGACAATTGGGATCTGCCCT	ATAGCTTCCGAGAGTGTTGTGGCA
*Tnfα*	TGTCTACTCCCAGGTTCTCT	GGGGCAGGGGCTCTTGAC
*Acta2*	CGATAGAACACGGCATCATC	CATCAGGCAGTTCGTAGCTC
*Mmp9*	CCCATGTCACTTTCCCTTCAC	GCCGTCCTTATCGTAGTCAGC
*Cers1*	CCACCACACACATCTTTCGG	GGAGCAGGTAAGCGCAGTAG
*Cers2*	ATGCTCCAGACCTTGTATGACT	CTGAGGCTTTGGCATAGACAC
*Cers3*	ATGGGCTTGTCTTCGTGAAAG	TTGCTTGTGGAATGCTTGAAAAA
*Cers4*	TACCCACATCAGACCCTGAAT	TGAAGTCCTTGCGTTTGACATC
*Cers5*	CGGGGAAAGGTGTCTAAGGAT	GTTCATGCAGTTGGCACCATT
*Asah1*	CGTGGACAGAAGATTGCAGAA	TGGTGCCTTTTGAGCCAATAAT
*Naaa*	GACTCCGCCTCTCTTCAACG	ACCATCCCGAGTACCCACTG
*B3galt4*	GGCAGTGCCCCTTCTGTATTT	CGAGGCATAGGGTGGAAAAG
*St3gal2*	CACCCTGACTCGGCTGCTT	TCTCGCGCCTTAGGGCTAA
Human		
*ACTB*	GCACAGAGCCTCGCCTT	GTTGTCGACGACGAGCG
*AURKA*	GCCCTGTCTTACTGTCATTCG	AGAGAGTGGTCCTCCTGGAAG
*AURKB*	ATCTGCTCTTAGGGCCAAGGG	CACATTGTCTTCCTCCTCAGGG
*NCD80*	TCAAGGACCCGAGACCACTTA	GGGAGCTTGTAGAGATTTCATGG
*TTK*	TGGCCAACCTGCCTGTTT	AATGCATTCATTTGCTGAAGAAGA
*TP53*	GGCCCACTTCACCGTACTAA	GTGGTTTCAAGGCCAGATGT

## Data Availability

The data that support the findings of this study are available from the corresponding author, upon reasonable request.

## Supplementary Materials

The following are available online at https://www.mdpi.com/2218-1989/11/3/167/s1, Figure S1: Gene expression of *Cers1* and *Cers2* in the livers of DDC or chow-fed mice; Figure S2: MALDI-TOFMS-based lipid imaging of the livers of DDC or chow-fed mice; Figure S3: CAGE-based transcriptome analysis of gene expression of ceramide metabolism in 50 parried of HCC tumors and non-tumor adjacent tissues; Figure S4: Score plot of PCA analysis on MALDI-TOFMS-based lipid profiling of JHH7 cells; Figure S5: Score plot of PCA analysis on nLC-MS/MS-based protein expression profiling of JHH7 cells; Figure S6: Upstream regulator analysis of AURKB using IPA; Table S1: Raw dataset of nLC-MS/MS-based proteome analysis; Figure S7: Western blot analysis of p53 and nuclear envelope marker Lamin B1 in the cytoplasmic and nuclear protein fractions of JHH7 cells treated with 25 μM PDMP for 24 h.
