# Supplementary figures and images for: Inhibition of Ganglioside Synthesis Suppressed Liver Cancer Cell Proliferation through Targeting Kinetochore Metaphase Signaling
Source: Metabolites. 2021 Mar 15;11(3):167. doi: 10.3390/metabo11030167 (PMC7998610; doi:10.3390/metabo11030167)

## Slide 1
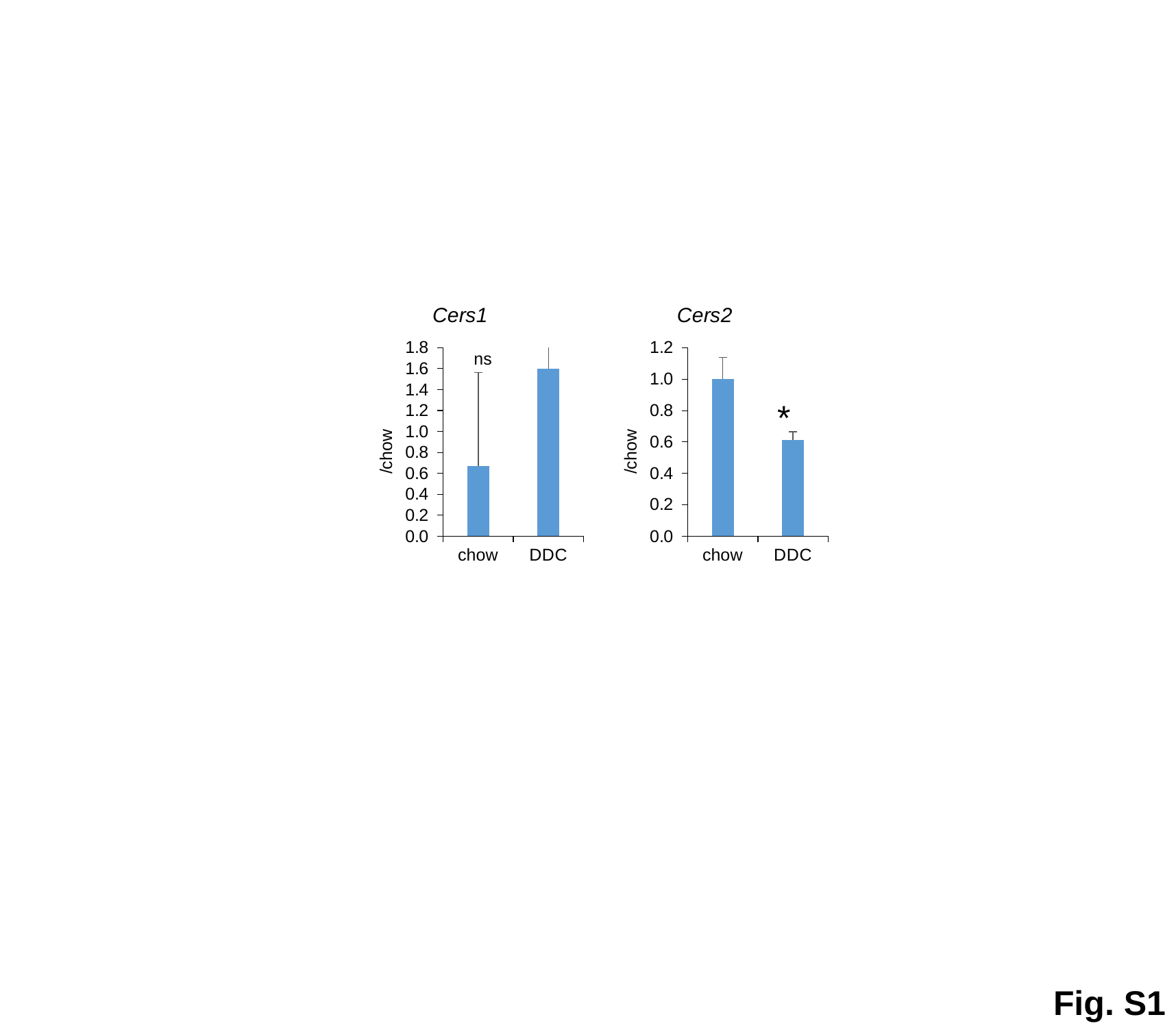

### Chart:
| Category | Cers2 |
|---|---|
| chow | 1.0 |
| DDC | 0.6106125612422841 |
### Chart:
| Category | Cers1 |
|---|---|
| chow | 0.6666666666666666 |
| DDC | 1.597682133305616 |ns
*
Fig. S1

Supplement: Supplementary file 1 [file metabolites-11-00167-s001.zip › Fig R1 S1.pptx]

## Slide 1
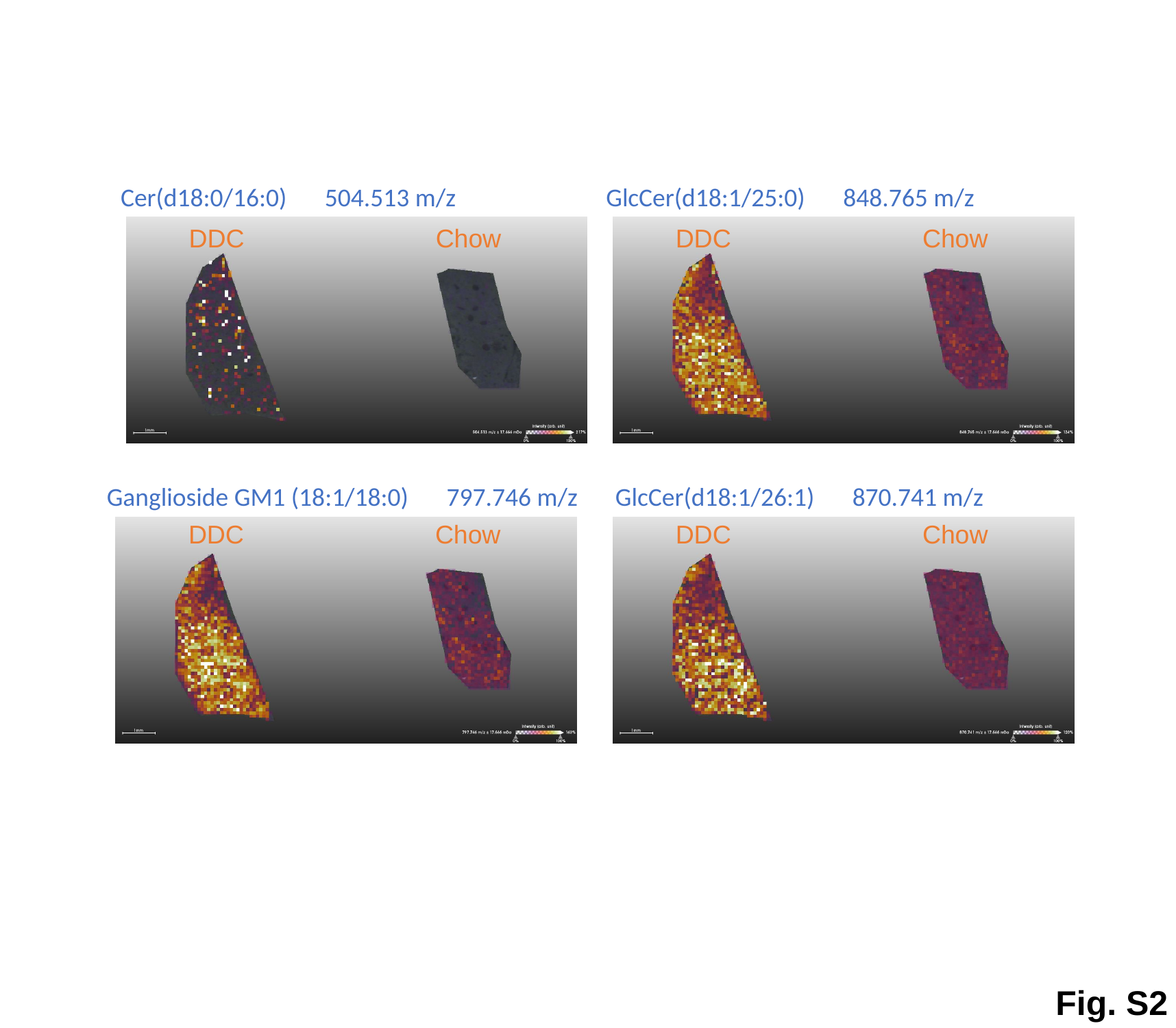

Cer(d18:0/16:0)　504.513 m/z
GlcCer(d18:1/25:0)　848.765 m/z
DDC
Chow
DDC
Chow
Ganglioside GM1 (18:1/18:0)　797.746 m/z
GlcCer(d18:1/26:1)　870.741 m/z
DDC
Chow
DDC
Chow
Fig. S2

Supplement: Supplementary file 1 [file metabolites-11-00167-s001.zip › Fig R1 S2.pptx]

## Slide 1
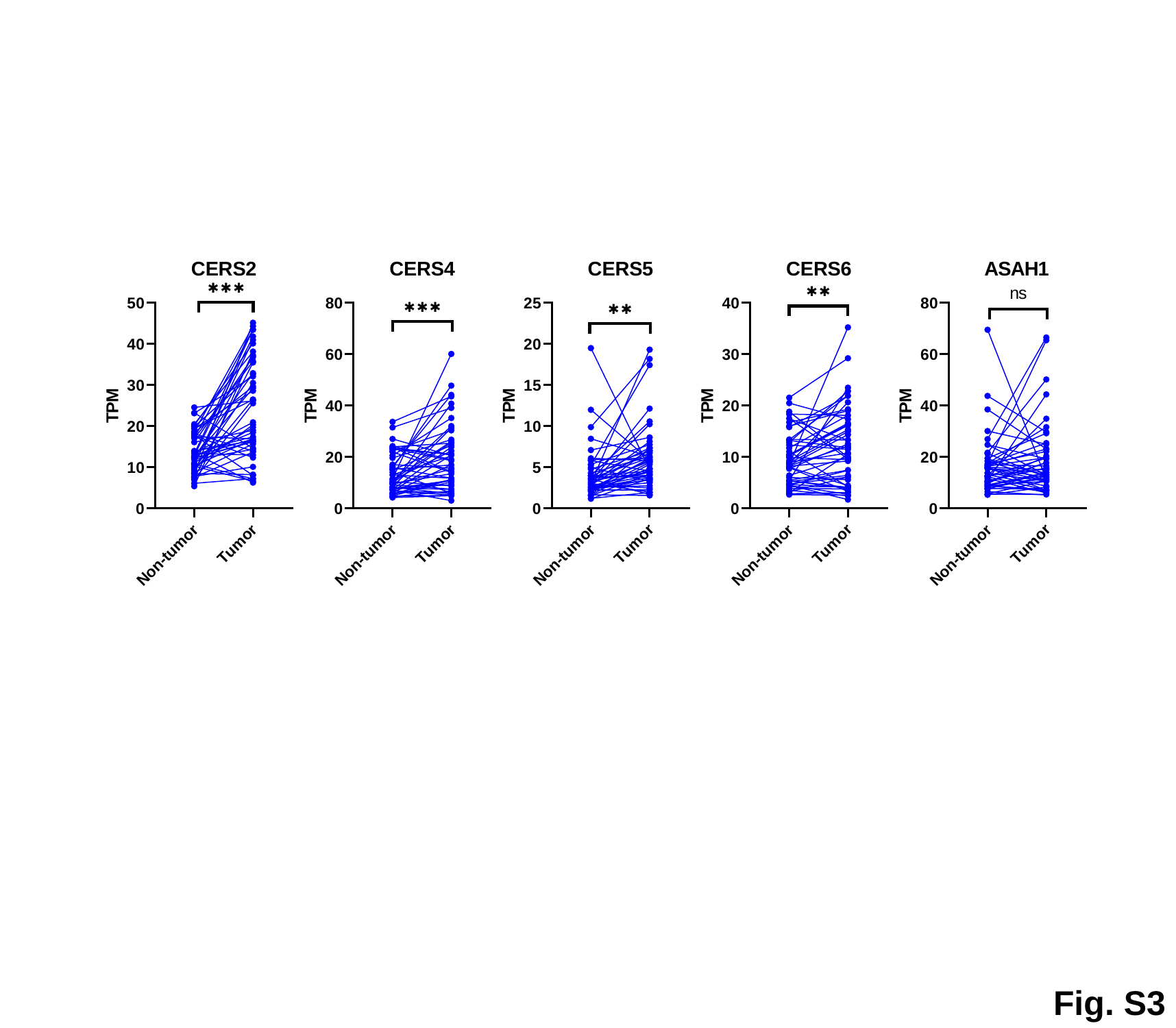

Fig. S3

Supplement: Supplementary file 1 [file metabolites-11-00167-s001.zip › Fig R1 S3.pptx]

## Slide 1
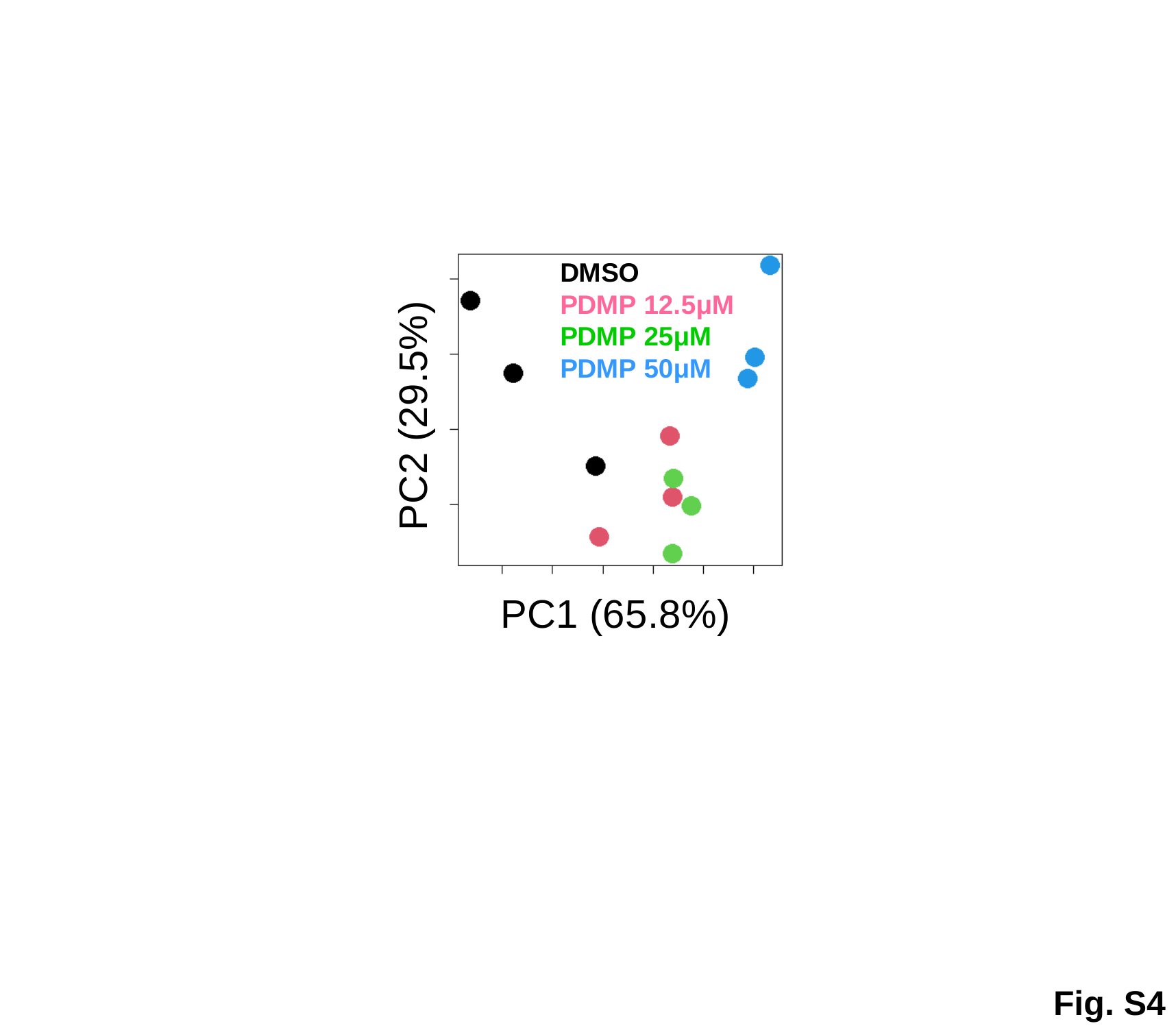

Fig. S4

Supplement: Supplementary file 1 [file metabolites-11-00167-s001.zip › Fig R1 S4.pptx]

## Slide 1
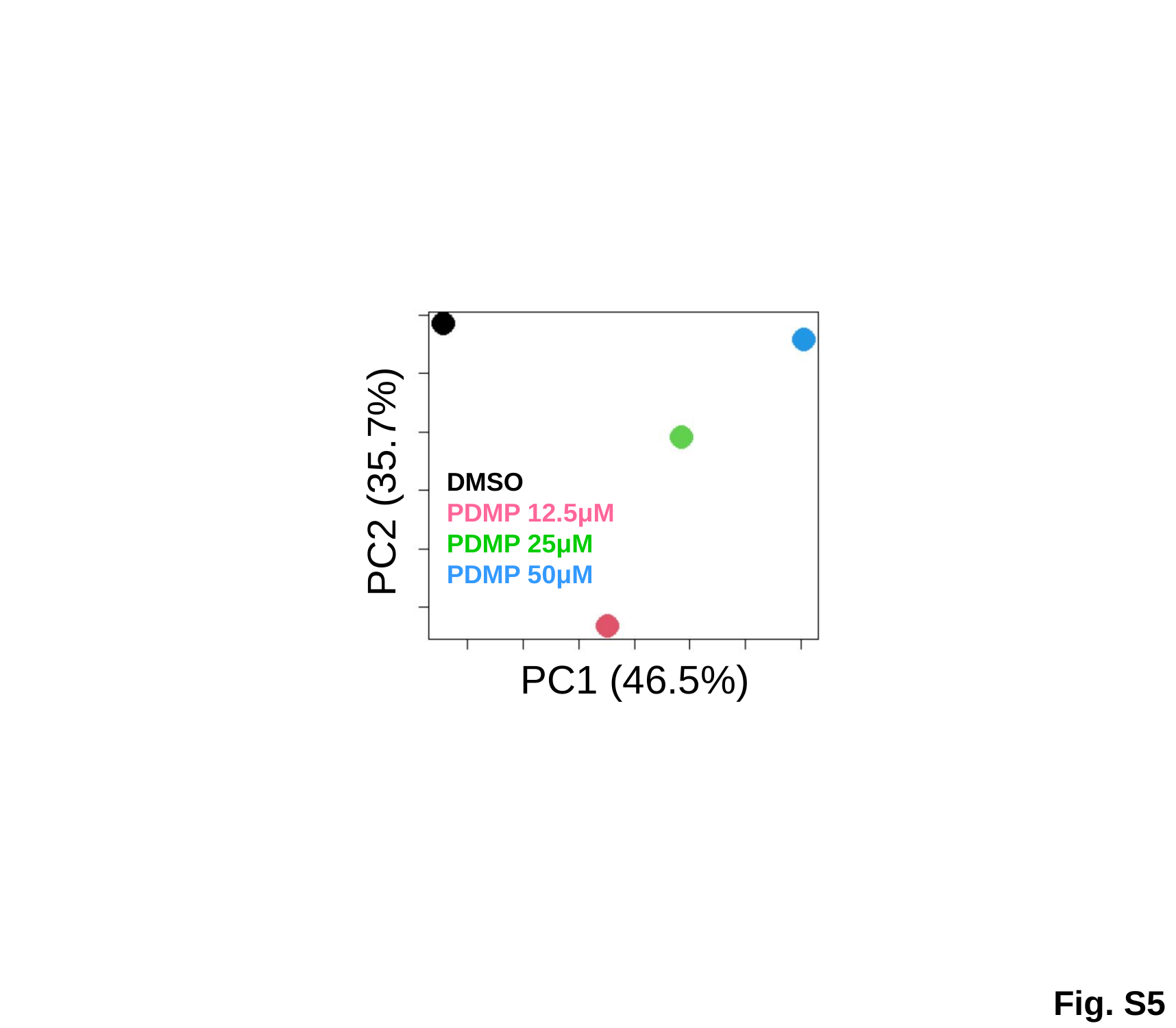

PC2 (35.7%)
DMSO
PDMP 12.5μM
PDMP 25μM
PDMP 50μM
PC1 (46.5%)
Fig. S5

Supplement: Supplementary file 1 [file metabolites-11-00167-s001.zip › Fig R1 S5.pptx]

## Slide 1
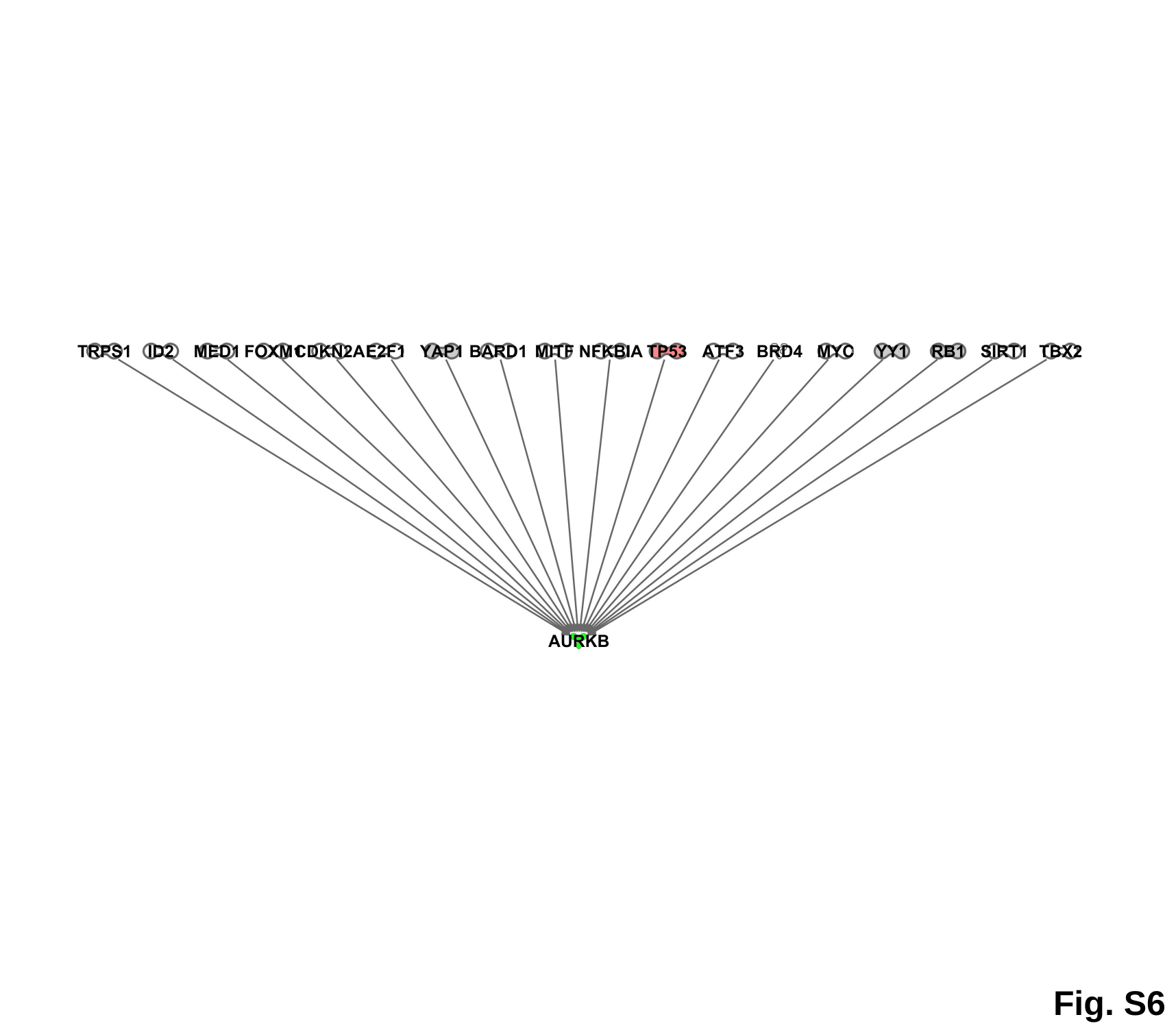

Fig. S6

Supplement: Supplementary file 1 [file metabolites-11-00167-s001.zip › Fig R1 S6.pptx]

## Slide 1
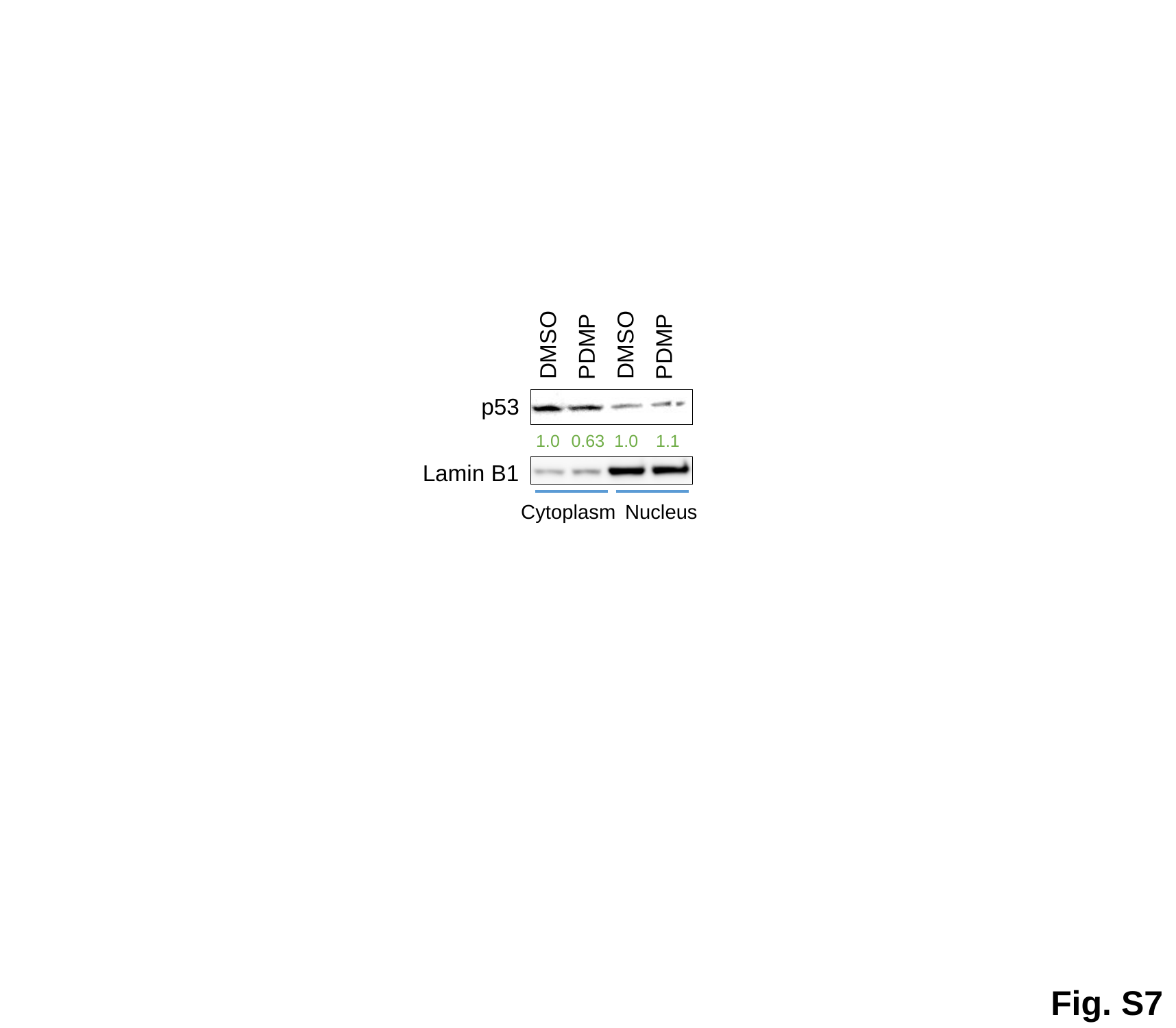

DMSO
DMSO
PDMP
PDMP
p53
1.0
0.63
1.0
1.1
Lamin B1
Cytoplasm
Nucleus
Fig. S7

Supplement: Supplementary file 1 [file metabolites-11-00167-s001.zip › Fig R1 S7.pptx]
